# A universal oral microbiome‐based signature for periodontitis

**DOI:** 10.1002/imt2.212

**Published:** 2024-06-12

**Authors:** Mingyan Geng, Min Li, Yun Li, Jiaying Zhu, Chuqing Sun, Yan Wang, Wei‐Hua Chen

**Affiliations:** ^1^ Institution of Medical Artificial Intelligence Binzhou Medical University Yantai China; ^2^ The Second School of Clinical Medicine Binzhou Medical University Yantai China; ^3^ Key Laboratory of Molecular Biophysics of the Ministry of Education, Hubei Key Laboratory of Bioinformatics and Molecular‐Imaging, Center for Artificial Intelligence Biology, Department of Bioinformatics and Systems Biology, College of Life Science and Technology Huazhong University of Science and Technology Wuhan China

## Abstract

We analyzed eight oral microbiota shotgun metagenomic sequencing cohorts from five countries and three continents, identifying 54 species biomarkers and 26 metabolic biomarkers consistently altered in health and disease states across three or more cohorts. Additionally, machine learning models based on taxonomic profiles achieved high accuracy in distinguishing periodontitis patients from controls (internal and external areas under the receiver operating characteristic curves of 0.86 and 0.85, respectively). These results support metagenome‐based diagnosis of periodontitis and provide a foundation for further research and effective treatment strategies.

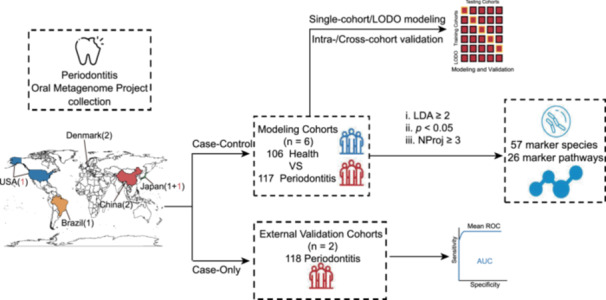

Periodontitis stands as one of the prevailing dental conditions, its prevalence steadily rising at a global age‐standardized incidence of 8.44% (6.62%–10.59%) from 1990 to 2019 [1]. Although certain genetic mutations predispose patients to higher disease risks, the pathogenic bacteria in the oral cavity, are believed to play important roles in the pathogenesis of the disease [2]. Notably, the species *Porphyromonas gingivalis*, *Tannerella forsythia*, and *Treponema denticola* are known as the “red complex.” They are the main pathogens of periodontitis and are positively correlated with the severity of periodontitis [3]. Significantly, patients suffering from severe periodontitis face heightened risks of contracting metabolic [4], respiratory diseases [5], cardiovascular diseases [6], and cancers [7]. Interestingly, this condition also exerts a considerable influence on the likelihood of developing neurological disorders [8]. The inflammatory processes and bacterial byproducts associated with periodontitis are believed to cross the blood−brain barrier, potentially triggering neuroinflammation and contributing to neural degeneration. Therefore, early diagnosis and prevention of periodontitis may have a potential predictive effect on the risk of aggravation of other diseases.

So far, stratification of periodontal patients from healthy controls can be achieved using gene‐, protein‐, and cytokine‐based biomarkers [9]. However, the success was limited because genetic factors could explain only a limited proportion of disease incidence, the flexibility of testing methods was limited, and the costs of related testing technologies and equipment were high [10]. Recently, researchers have successfully developed machine learning (ML) models based on the oral microbiome of periodontitis patients obtained from 16S amplicon sequencing. For example, Huang et al. employed two cohorts to construct a genus‐level classifier for distinguishing periodontitis states. They developed a feature selection algorithm based on random forests to classify healthy and periodontitis groups, achieving an average area under the receiver operating characteristic curve (AUC) of 0.80 [11]. However, the study suffered from low taxonomic resolution, limited data sets, and a lack of a wider representation of the global population. A comprehensive species‐level evaluation using multiple cohorts is warranted to understand shared and study‐specific oral microbiome alterations in periodontitis, as well as high‐performance and robustness ML models building across cohorts.

## RESULTS AND DISCUSSION

### Collection and analysis of oral metagenome data sets for periodontitis

**Figure 1:**
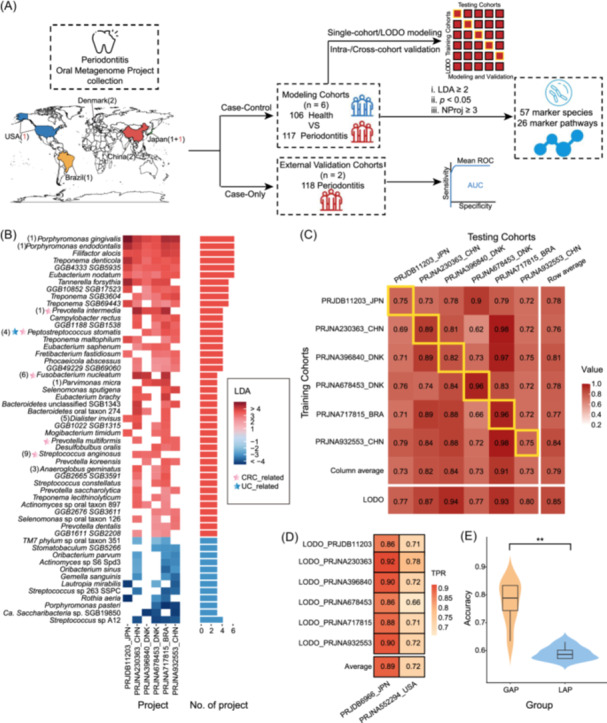
Identification of cross‐cohort biomarkers and performance of oral microbiome‐based classifier across multiple periodontitis cohorts. (A) Flowchart shows the global distribution of the eight public data sets and the overview of our data analysis procedure, including biomarker identification, intra‐ and cross‐cohort modeling, and validation. Among the eight cohorts, six contained both patients and controls (case‐control cohorts), indicated with black numbers on the map, while two patient‐only cohorts are indicated with red regular numbers. (B) Heatmap‐bar plot shows the distribution of biomarkers that were significantly differentially distributed in at least three projects according to Linear discriminant analysis effect size (LEfSe) on MetaPhlAn4 species profiles. The red blocks in the heatmap indicate disease‐enriched and blue blocks indicate health‐enriched. Pink and blue stars at the species names indicate species related to the risk of colorectal cancer (CRC) and Crohn's disease (CD), respectively, according to Jiang et al. [12]. The number in parentheses before the species name corresponds to the number of diseases in which the biomarkers were enriched, according to the GMrepo database [13] (Table S4). (C) Cross‐prediction matrix reporting prediction performances as AUC values obtained using a Random Forest (RF) model on species‐level relative abundances. The values boxed in yellow squares on the diagonal are the AUC values obtained by training and validating within individual cohorts. The nondiagonal values refer to training a classifier on the data set corresponding to the row and applying it to the data set corresponding to the column to obtain the AUC value. The row average and column average are the average values of excluding the diagonal values. The LODO row refers to the performances obtained by training the model on the species‐level abundances using all but the data set of the corresponding column and applying it to the data set of the corresponding column. Color gradient represents the value of AUCs. (D) True positive rates (TPRs) of the LODO models on the external two case‐only cohorts. (E) Effects of disease severity on the performance of the LODO models. Samples in PRJNA552294_USA were divided into two groups, namely generalized aggressive periodontitis (GAP; more severe) and localized aggressive periodontitis (LAP; less severe). Significance was determined using the Wilcoxon rank‐sum test (two‐sided), **p ＜0.01. AUC, area under the receiver operating characteristic curve; LDA, linear discriminant analysis; LODO, leave‐one‐data set‐out analysis; Nproj, the number of projects.

To identify oral microbiome signatures of universal representativeness for periodontitis, we systematically collected eight publicly available shotgun metagenomic sequencing projects that were from populations of five countries across three continents (Figure 1A). Among these, six included both case and control groups and were used for modeling analyses, with 117 disease samples and 106 healthy controls. The other two case‐only projects were used for further validation, with 118 disease samples (Figure S1 and Tables S1 and S2).

We observed significantly higher microbial community diversities within the periodontitis samples compared to those within the healthy samples in three out of the six projects (Figure S2A), represented by Shannon indexes, consistent with previous results that the inflammation in the periodontal pockets can lead to higher bacterial loads and increased bacterial diversity [14]. Additionally, we observed significantly different community structures of the periodontitis patients as compared to the controls in all but one project (Figure S2B), as indicated by the principal coordinates analysis (PCoA). These results collectively indicate distinct oral microbiome community characteristics between periodontitis and the controls.

### Identification of cross‐cohort species biomarkers for periodontitis

We next sought to identify microbial biomarkers that showed consistent trends across cohorts. Among a total of 238 species that showed significant abundance changes in at least one cohort, we identified 54 that showed consistent trends in three or more cohorts (Figure 1B and Table S3), including 42 and 12 disease‐ and health‐enriched ones, respectively (Figure 1B). The disease‐enriched markers included many known periodontitis‐associated species, including the “red complex” members (i.e., *P. gingivalis*, *T. forsythia*, and *T. denticola*), and *Porphyromonas endodontalis*, *Filifactor alocis*, and *Eubacterium nodatum* [3]. All were identified as biomarkers in all six cohorts, except *T. forsythia*, which was enriched in five cohorts. Additionally, we also identified novel species that have not been widely studied, including *GGB4333 SGB5935*, which was enriched in six cohorts, and three novel species present in five cohorts, which should be experimentally validated in the future. This uniformity not only facilitates early detection but also refines treatment precision, which is instrumental in improving patient outcomes. In clinical practice, these biomarkers are potentially useful in distinguishing periodontitis from controls and design of polymerase chain reaction (PCR)‐based rapid detection methods for risk evaluation before the disease presents prominently. It can be applied to the occasion of physical examination and screening for early‐onset people without obvious symptoms as a supplement to clinical diagnosis.

Periodontitis‐related oral bacteria are known to translocate to the gut and exacerbate gut inflammation [15]. We found that some of these biomarkers were also enriched in the gut microbiome in patients of colorectal cancer (CRC) and Crohn's disease (CD) (Figure 1C), according to the GMrepo database [13] (Figure S3A and Table S4). In part due to an increase in oral microbial abundances in the gut microbiome of these diseases [16].

We also identified 12 health‐enriched cross‐cohort markers. Notably, *Streptococcus* sp. A12 was abundant in the healthy samples (Figure S3B), helping maintain the stability of the oral biofilm by inhibiting the growth of *Streptococcus mutans*, thereby reducing harmful bacteria growth [17]. These health‐enriched biomarkers could be key to sustaining a healthy oral microbiome and may serve as targets for future interventions.

### Taxonomy‐based classifiers have high classification performance and cross‐cohort generalization ability

To test whether the taxonomic relative abundance of the oral microbiome can be used to distinguish cases from controls in each cohort, we first built a random forest classifier using all species features and validated its performance using five‐fold three times repeated intra‐cohort cross‐validation. We achieved an average internal AUC of 0.86 with an interquartile range (IQR) of 0.77–0.94 (mean along the diagonal; Figure 1C). The classifier also performed well externally, with an average AUC of 0.79 (IQR: 0.73–0.83, Figure 1C; off‐diagonal column average) and all models had an average external AUC higher than 0.75 (off‐diagonal row average), indicating its effectiveness across different cohorts.

Using leave‐one‐data‐set‐out (LODO) analysis, we enhanced our model's ability to generalize across cohorts, raising the external mean AUC from 0.79 to 0.85 (IQR: 0.785–0.900, Figure 1C). The improvement could be observed for each cohort (Figure 1C, the bottom row of the heatmap).

We further tested the robustness of our LODO classifiers on two additional patient‐only cohorts and achieved average true positive rates (TPRs) of 89% (*n* = 50, PRJDB6966) and 72% (*n* = 68, PRJNA552294), respectively (Figure 1D). The slightly lower TPR of the later cohort could be attributed to mixed patients with varied disease severity. After separating the patients into different groups as suggested by Eija Könönen et al. [18], we obtained TPRs of nearly 80% in the generalized aggressive periodontitis (GAP, *n* = 34) group and nearly 60% in the localized aggressive periodontitis (LAP, *n* = 34) group (Figure 1E), indicating that our models are exceptionally precise in identifying the server patients, while retaining appreciable sensitivity for those with minor symptoms. Consequently, these models hold promise for facilitating early risk assessment and intervention strategies.

To estimate the minimal sample size for our classifiers, we did a sample‐cumulation modeling (SCM) [19] and found that only 40 samples (i.e., 20 cases and 20 controls; Figure S4A) were needed to obtain external validation AUCs of 0.81, far below the total number of samples in our six case‐control cohorts (117 patients and 106 controls; Tables S1 and S2), suggesting adequate sample size for robust model training in this study. In the future, the robustness of the classifiers should be tested in clinical trials and longitudinal studies with larger cohorts and at multiple centers.

We also examined the important features ranked by the ML classifiers and found that *F. alocis* was ranked as the top case‐enriched species, followed by other well‐known species like *Fusobacterium nucleatum*. Most of the top‐ranked species overlapped with the biomarkers identified in the previous section, which are indicated by red and blue stars in Figure S4B.

### Network analysis reveals predominantly positive interactions among biomarkers of health‐ or disease‐groups

**Figure 2:**
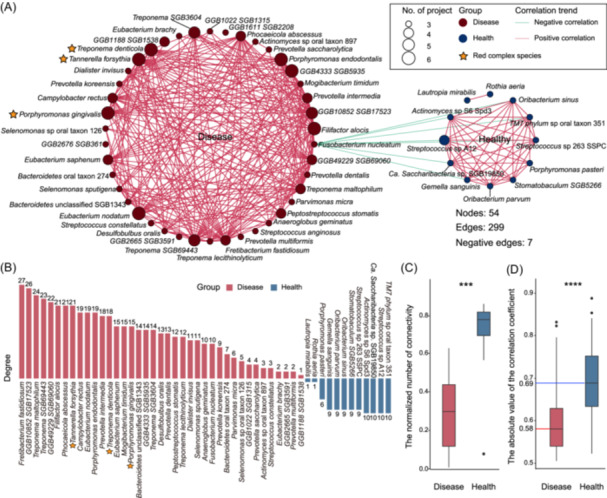
Network analysis of cross‐cohort microbial biomarkers. (A) A network plot shows the correlations within and between the 12 health‐enriched and 42 disease‐enriched species that had significant Spearman Correlation coefficients (i.e., with |correlation coefficient | > 0.5 and *p* value < 0.05). The size of the nodes varied with the number of projects. Species names prefixed with stars are members of the “red complex.” (B) The bar plot shows the network connectivity statistics of the health‐ and disease‐enriched species among their respective groups. (C) Box plot shows the normalized network connectivity of the biomarkers in their respective groups. Significance was determined using the Wilcoxon rank‐sum test (two‐sided), ***p < 0.001. (D) Box plot shows the absolute correlation coefficients among the disease‐ and health‐biomarkers in their respective groups. Two‐sided Wilcoxon rank‐sum test was used for group‐wise comparisons, ****p < 0.0001. The healthy cohort was colored in blue and the disease cohort was colored in red.

We next explored the interplays among the aforementioned biomarkers species using correlation network analysis. Applying an absolute Spearman correlation coefficient (SCC) threshold > 0.5 and a *p* < 0.05, we identified a total of 299 interactions among the cross‐cohort biomarkers (Figure 2A and Table S5), with a majority of being positive correlations within the health or disease groups. Specifically, we identified 43 positive correlations among the 12 health‐enriched species and 249 pairs among the 42 disease‐enriched species. Notably, a novel species, *GGB10852 SGB17523*, mainly cooperated with disease‐enriched species in the interaction network and the degree ranked second among all biomarkers (Figure 2B and Table S6), indicating its potential role in periodontitis. Although disease‐biomarkers exhibited significantly higher within‐group connectivity than that of health‐biomarkers, they displayed significantly lower normalized within‐group connectivity scores (i.e., connectivity divided by the number of biomarkers in the group) than that of the health‐biomarkers (Figure 2C). Additionally, disease‐biomarkers demonstrated significantly weaker correlations (median SCC of 0.58) than that of health‐biomarkers (median SCC of 0.69) (Figure 2D). This indicates that a higher synchronization among health biomarkers may adapt to changes and stresses in the oral environment, supporting the balance and stability of oral ecology.

Conversely, we observed very few interactions (seven negative interactions) between members of the health‐ and disease‐biomarkers (Figure 2A). Among these, *F. nucleatum* of the disease group was the only one showing correlations with the health‐group members. This suggests that supplementing individual health‐biomarkers, as Zhang et al. have demonstrated [20], might not be an effective treatment strategy for developing a comprehensive approach to enhance the physiological conditions of the oral cavity, favoring the growth of health‐biomarkers while suppressing disease‐biomarkers.

### Function‐based classifiers have low classification performance for periodontitis

We also sought to assess the oral microbiota's functions in periodontitis and their ability to differentiate patients from controls based on pathway abundance. We observed no significant differences between the case and control group in alpha diversity analysis in all but one cohort (Figure S5A), although four out of six projects had significantly different community structures using beta diversity analysis (Figure S5B). These results implied a limited capacity for functional features rather than taxonomic features in distinguishing between the case and control. Consistently, we identified fewer consistently altered metabolic pathways (*n* = 26) in three or more cohorts (Figure S5C and Table S7) as compared with the species biomarkers (*n* = 54).

Among the case‐enriched pathways, two involving histidine degradation (i.e., histidine degradation III and I) were ranked first and second, respectively, and they were the only two biomarkers enriched in more than five cohorts (Figure S5C). Additional case‐enriched pathways included responses related to one‐carbon metabolism or stress responses, indicating that the oral microbiota is subject to oxidative stress or immune challenges in disease states, necessitating adjustments in metabolic equilibrium and protective mechanisms. Among the control‐enriched pathways, there is a higher synthesis capability of amino acids, nucleotides, and vitamins (Figure S5C) in the oral microbiota under healthy conditions, potentially providing essential or beneficial compounds to the host.

We also built ML classifiers using the functional profiles, but achieved moderate success in distinguishing cases from controls, with an average intra‐cohort AUC of 0.755 (Figure S5D; values along the diagonal). However, these classifiers had limited success on external cohorts, with a lower average external AUC of 0.58. The LODO classifiers analysis slightly improved performance, with an average external AUC of 0.64 (Figure S5D), suggesting the need for more consistent metabolic biomarkers for better generalization.

Our final tests showed that classifiers using both taxonomic and functional data did not outperform those using taxonomic data alone, as external AUCs were significantly lower (Figure S6). Consequently, we chose the taxonomy‐based model for classification.

## CONCLUSION

We have comprehensively evaluated the oral microbiome‐based signatures for periodontitis, demonstrating its universality and robustness across eight globally distributed cohorts. Our results strongly support the feasibility of a metagenome‐based diagnosis of periodontitis and lay the foundations for further mechanistic research and the development of effective diagnostic, treatment, and intervention strategies.

## AUTHOR CONTRIBUTIONS

Wei‐Hua Chen and Yan Wang designed and directed the research. Mingyan Geng and Min Li analyzed the data, performed modeling, and wrote the paper with results from all authors. Yun Li, Jiaying Zhu, and Chuqing Sun helped with data collection and figure refinement. Wei‐Hua Chen and Yan Wang polished the manuscript through multiple iterations of discussions with all authors. All authors have read the final manuscript and approved it for publication.

## CONFLICT OF INTEREST STATEMENT

The authors declare no conflict of interest.

## ETHICS STATEMENT

No animals or humans were involved in this study.

## Supporting information


**Table S1:** Information of the collection data sets.
**Table S2:** Metadata for the samples in detail.
**Table S3:** Details of species markers.
**Table S4:** Marker species‐related disease according to the Gmrepo database.
**Table S5:** Results of network analysis.
**Table S6:** Network connectivity statistics of the health‐ and case‐enriched species.
**Table S7:** Details of pathway markers.
**Table S8:** Sequencing depth of all the samples.

**Figure S1:** The pipeline for metagenomics data collection.
**Figure S2:** Species diversity was analyzed based on taxonomic relative abundance data.
**Figure S3:** Disease types with biomarkers enriched as well as the abundance.
**Figure S4:** Trends in external AUC with several training samples and heatmap of species ranked by RF classifiers.
**Figure S5:** Function‐based diversity analysis, identification of cross‐cohort pathway biomarkers, and modeling of machine learning classifiers for periodontitis.
**Figure S6:** Prediction performances of the models based on the combined taxonomic‐functional profiles in within‐cohort validation and cross‐cohort testing.
**Figure S7:** Evaluation of batch‐effect removal tools on the six case‐control projects.
**Figure S8:** The performance of four machine learning algorithms in cross‐validation.

## Data Availability

Metagenome sequencing data can be freely accessed in NCBI Sequence Read Archive (SRA) under the study accession number PRJDB11203 (https://www.ncbi.nlm.nih.gov/bioproject/?term=PRJDB11203), PRJNA230363 (https://www.ncbi.nlm.nih.gov/bioproject/?term=PRJNA230363), PRJNA396840 (https://www.ncbi.nlm.nih.gov/bioproject/?term=PRJNA396840), PRJNA678453 (https://www.ncbi.nlm.nih.gov/bioproject/?term=PRJNA678453), PRJNA717815 (https://www.ncbi.nlm.nih.gov/bioproject/?term=PRJNA717815), PRJNA932553 (https://www.ncbi.nlm.nih.gov/bioproject/?term=PRJNA932553), PRJDB6966 (https://www.ncbi.nlm.nih.gov/bioproject/?term=PRJDB6966), PRJNA552294 (https://www.ncbi.nlm.nih.gov/bioproject/?term=PRJNA552294). The data and scripts used are saved in GitHub (https://github.com/whchenlab/Oral-Microbiome-Based-Signature-for-Periodontitis). Supplementary materials (methods, figures, tables, scripts, graphical abstract, slides, videos, Chinese translated version, and update materials) may be found in the online DOI or iMeta Science http://www.imeta.science/.
