# Supplementary material for: A universal oral microbiome‐based signature for periodontitis
Source: Imeta. 2024 Jun 12;3(4):e212. doi: 10.1002/imt2.212 (PMC11316925; doi:10.1002/imt2.212)
Supplement: Supplementary file 2 — Figure S1: The pipeline for metagenomics data collection. Figure S2: Species diversity was analyzed based on taxonomic relative abundance data. Figure S3: Disease types with biomarkers enriched as well as the abundance. Figure S4: Trends in external AUC with several training samples and heatmap of species ranked by RF classifiers. Figure S5: Function‐based diversity analysis, identification of cross‐cohort pathway biomarkers, and modeling of machine learning classifiers for periodontitis. Figure S6: Prediction performances of the models based on the combined taxonomic‐functional profiles in within‐cohort validation and cross‐cohort testing. Figure S7: Evaluation of batch‐effect removal tools on the six case‐control projects. Figure S8: The performance of four machine learning algorithms in cross‐validation. [file IMT2-3-e212-s002.docx]

# Supporting information to

## A Universal Oral Microbiome-Based Signature for Periodontitis

**Running title**: Oral Microbiome Signature for Periodontitis

Mingyan Geng^1, 2, 3#^, Min Li^3#^, Yun Li^3^, Jiaying Zhu^3^, Chuqing Sun^3^, Yan Wang^2, 3*^, Wei-Hua Chen^3*^

^1^The Second School of Clinical Medicine, Binzhou Medical University, Yantai 264003, China

^2^Institution of Medical Artificial Intelligence, Binzhou Medical University, Yantai 264003, China

^3^Key Laboratory of Molecular Biophysics of the Ministry of Education, Hubei Key Laboratory of Bioinformatics and Molecular-imaging, Center for Artificial Intelligence Biology, Department of Bioinformatics and Systems Biology, College of Life Science and Technology, Huazhong University of Science and Technology, Wuhan 430074, China

^#^These authors contributed equally: Mingyan Geng, Min Li

*Correspondence: weihuachen@hust.edu.cn (Wei-Hua Chen) or yanw@hust.edu.cn(Yan Wang)

# Methods

### Data collection

We searched for periodontitis-related oral microbiome datasets in the NCBI BioProject (<https://www>.ncbi.nlm.nih.gov/bioproject) database using the following keyword combinations: “(‘human metagenome’[Organism] OR human metagenome[All Fields]) AND oral[All Fields]” and “(‘human oral metagenome’[Organism] OR human oral metagenome[All Fields])”; please consult Figure S1 for the selection process and results.

Our collection criteria includes:

1. Case-control studies with clear disease information: This means that we only select studies where cases have been diagnosed with periodontitis disease, and healthy controls without periodontitis;
2. At least 10 valid samples in each case group and control group: This ensures that our results have sufficient stability and reproducibility;
3. No recent use of antibiotics: Antibiotic use may affect the composition of oral microbiota, thereby affecting the accuracy of our research.

After filtering out duplicates and data without detailed metadata or meeting minimum samples requirements, we selected a total of eight datasets. Among these, six datasets including both case and control were used for machine learning (ML) modeling and validation, and two datasets with only patient samples were used for independent testing of the ML models. The project accession numbers used for modeling include PRJDB11203, PRJNA230363, PRJNA396840, PRJNA678453, PRJNA717815, and PRJNA932553, and the project accession numbers used for external validation include PRJDB6966 and PRJNA552294 (Table S1 and S2).

### Raw metagenomic data processing

The raw sequencing data were downloaded from the NCBI Sequence Read Archive (SRA) database (<https://www.ncbi.nlm.nih.gov/sra>) [1]. We used Trimmomatic [2] (v.0.39) with TruSeq3 adapter files to remove adapter sequences and low-quality reads from the raw data. The TruSeq3-PE.fa file was used for paired-end sequences, while the TruSeq3-SE.fa file was used for single-end sequences. Reads shorter than 50bp were removed. Since our data was generated through metagenomic next-generation sequencing (mNGS), we utilized Bowtie2 [3] (v.2.5.1) to align the filtered reads to the human genome (hg19) to remove human DNA contaminations. The remaining reads were referred to as “clean data” and were used for subsequent analyses. Please consult Supplementary Table S2 for a complete list of projects, samples, and run IDs used in this study.

### Taxonomic and functional profiling

We used the MetaPhlAn4 [4] (v.4.0.3) software with default parameters for taxonomic analysis and retained the relative abundances at the species level for subsequent analysis. For the functional profiles including metabolic pathways, we used the HUMAnN3 [5] (v.3.6) software with default parameters.

To avoid the impact of lowly abundant taxonomic and functional entities, we filtered the species abundance spectrum and metabolic pathway abundance spectrum within each project, removing species and pathways with maximum relative abundance lower than 0.001 in all samples of the project, according to Li *et al.* [6] and the reference manual of “SIAMCAT” R package [7] (v.1.9.0, <https://bioconductor.org/packages/SIAMCAT>). The filtered microbial abundance data and metabolic pathway abundance data were z-score standardization for subsequent modeling and statistical analysis.

### Identification and removal of confounding factors within cohorts

Due to the influence of confounding factors, subsequent analyses may introduce bias. These issues can potentially compromise the reliability and effectiveness of subsequent biomarker identification and disease prediction modeling. To avoid these problems, we identified confounding factors for each project and subsequently eliminated their impact on the taxonomic and functional relative abundance profile to ensure the quality of results. More specifically, we examined all available factors in the metadata, such as age, gender, body mass index (BMI), disease stage, and geographic location. We tested for significant differences between the case group and the control group. For qualitative variables (including age and BMI), we used the Fisher‘s exact test, while for quantitative variables (including gender, disease stage, and geographic location), we used the non-parametric Wilcoxon rank sum test. We then used the “removeBatchEffect” function from the “limma” R Package [8] (v.3.56.0) to adjust the factors with *p* < 0.05. Significant qualitative and quantitative variables were considered as covariates and batch factors, respectively, with other variables set as default.

### Batch effect removal across cohorts

We evaluated the effectiveness of the state-of-art batch removal methods, including the “ComBat” function in the “sva” R package [9] (v.3.48.0), “removeBatchEffect” in the “limma” R package [8] (v.3.56.2), “adjust_batch” in the “MMUPHin” R package [10] (v.1.14.0), and “ConQuR” in the “ConQuR” R package [11] (v.2.0) that are widely used in current microbiome research. The batch effect was quantified based on the R-squared value from PERMANOVA testing and the distribution in PCoA plots (Figure S7). The smaller the R-square value, the smaller the proportion of batch effects in the model and the less impact it has on our model construction. Based on this criterion, we selected and employed the “adjust_batch” function in the “MMUPHin” R package (v.1.4.2) to reduce batch effects, using project ID and shared confounding factors as control factors. After removing confounding or reducing batch effects, subsequent modeling and biostatistical analysis were conducted on relative abundance data.

### Biomarker identification using Linear discriminant analysis Effect Size (LEfSe)

We used the “run.lefse” function in the “microbiomeMarker” R package [12] (v.1.0.2) to perform LEfSe analysis to identify disease-specific taxa. The output of the LefSe analysis provides effect size scores, which represent the Linear Discriminant Analysis (LDA) scores, reflecting the degree of difference between case and control groups. The higher the score, the more significant the difference. Taxa with *p* <= 0.05 and LDA score of 2 in at least one cohort or greater were considered biomarkers. In this study, we added a plus (+) or minus (-) sign to the score to indicate their enrichment in the case or control group, respectively. We extracted biomarkers and metabolic pathways that were enriched in three or more projects for further validation analysis and mapping.

### Microbial network analysis

To characterize the relationships among the marker species and the resulting interaction networks, we used the spearman method in the “corr.test” function of the “psych” (v.2.3.9, [https://CRAN.R-project.org/package=psych](https://cran.r-project.org/package=psych)) package to calculate the correlation. Correlations with a *p* < 0.05 and an absolute value of the correlation coefficient > 0.5 were retained for further analysis. We used the “ggraph” package (v.4.3.2, <https://CRAN.R-project.org/package=ggraph>) to visualize the network, with the size of the nodes corresponding to the number of the projects, and positive and negative correlation edges painted green and red, respectively.

The normalized number of health-enriched species connectivity and disease-enriched species represent the ratio of each health-enriched species divided by the total number of health-enriched species (12) and each disease-enriched species divided by the total number of disease-enriched species (42). Two-sided Wilcoxon rank-sum test was used for group-wise comparisons.

### Machine Learning Modeling, Validation and Testing

To check whether oral microbiome data can be used to distinguish periodontitis from healthy controls, we used the “SIAMCAT” R package [7] (v.1.9.0, <https://bioconductor.org/packages/SIAMCAT>) to construct disease grading classifiers (or models). Related abundances were normalized using the “normalize.features” function and then used as input for model training, validation, and testing.

To select the best machine learning algorithm, we compared four methods including Elastic Net (Enet) [13], Lasso [14], Random Forest (RF) [15], and Ridge Regression (Ridge) [16] that corresponded to the parameters “lasso”, “enet”, “ridge”, “randomForest” respectively in the “train.model” function of the SIAMCAT package. By plotting a boxplot, we observed that there was no significant difference in modeling between the four methods. However, the results of the random forest modeling were the best (Figure S8). Therefore, we chose random forest as our machine learning algorithm.

The num.folds and num.resample parameters in the “create.data.split” function were used to adjust for different datasets, including intra-cohort (num.folds = 5,num.resample = 3) and combined cohort (num.folds = 10,num.resample = 3) modeling and validation. The model was then established using the “train_model” function. The “make.predictions” and “evaluate.predictions” functions were used for prediction. The “pROC” R package [17] (v.1.18.5) was then used to calculate the area under the receiver operating characteristic curve (AUC) score as a measure of prediction performance. By default, all features were used for model training and validation, as recommended by refs [7, 18].

In addition to training models on individual datasets, leave-one-dataset-out (LODO) analyses [19] were also performed, which involved training a classifier model on n-1 datasets and validating it on the remaining one dataset at a time when there were at least three datasets available [20]. The LODO analysis examines whether incorporating multiple cohorts for model training can improve the predictive performance of classifiers, in order to test whether the cross-validation model is biased towards a specific dataset.

In this study, we also employed the Sample-Cumulation Modeling (SCM) approach to determine the relationship between sample sizes and AUC values as a combined-cohort modeling and validation strategy [6]. For the above combined-cohort modeling(LODO and SCM), we used ten folds three times repeated cross-validation (num.folds = 10, num.resample = 3 in the create.data.split function) and only noted external (cross-cohort) validation AUCs. The SCM analysis examines whether the model performance increases with the increasing number of samples in modeling training.

### Statistical analysis and bioinformatics methods

All processed data, if not specifically stated, were loaded into R (version 4.1.2, <https://www>.r-pro ject.org/) for analysis and visualization. Wilcoxon rank sum test was used for two-group comparison , while the Kruskal-Wallis test was used for multi-group comparisons, using the “ggpubr” R package (v.0.4.0, <https://github>.com/kassambara/ggpubr) in “stat_compare_means” function with default parameters. When performing multiple hypothesis tests, the corrected Wilcoxon rank test was performed by the “ggpubr” package “compare_means” function with default parameters. The correlation analysis used the spearman correlation test. All tests were two-sided with *p* < 0.05 (when two groups are compared) or a corrected *p* of < 0.05 (when multiple groups are compared) considered statistically significant. We used the diversity function in the “vegan” R package [21] (v2.6.4) to calculate the alpha diversity of each dataset using the Shannon diversity index and abundance coverage estimator (ACE), which queries species diversity and abundance. We used the Bray-Curtis index to calculate the differences between samples and estimate beta diversity. We perform PCoA analysis and visualization using the “pcoa” function in the ape R package [22] (v.5.7-1).

# Supplementary Figures


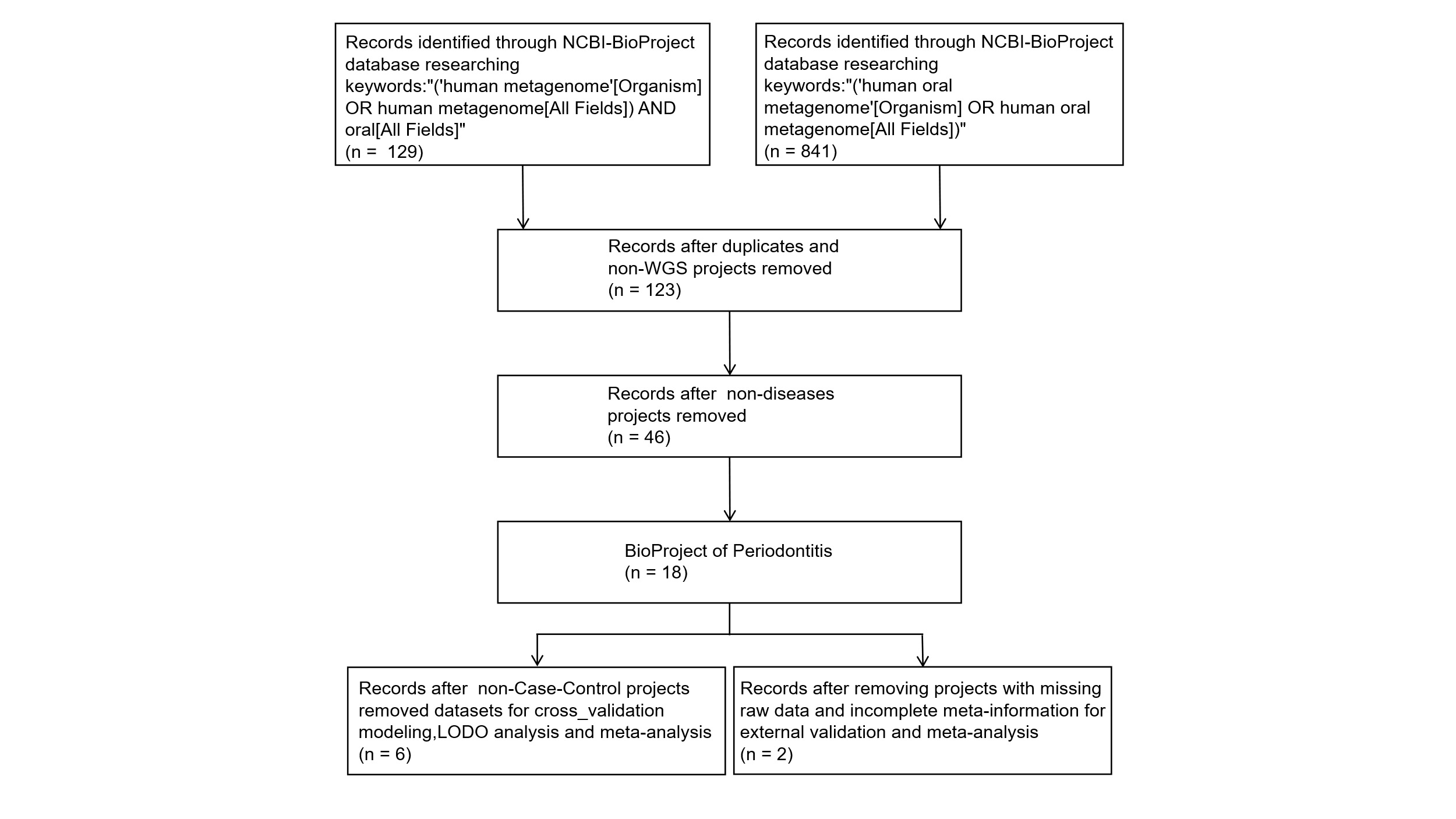


**Figure S1 The pipeline for metagenomics data collection.** We collected records about periodontitis in public databases until September 2023. The requirements for samples were that (i)at least 10 valid samples in each case group and control group, and (ii) there were no overlapping samples across datasets.

**
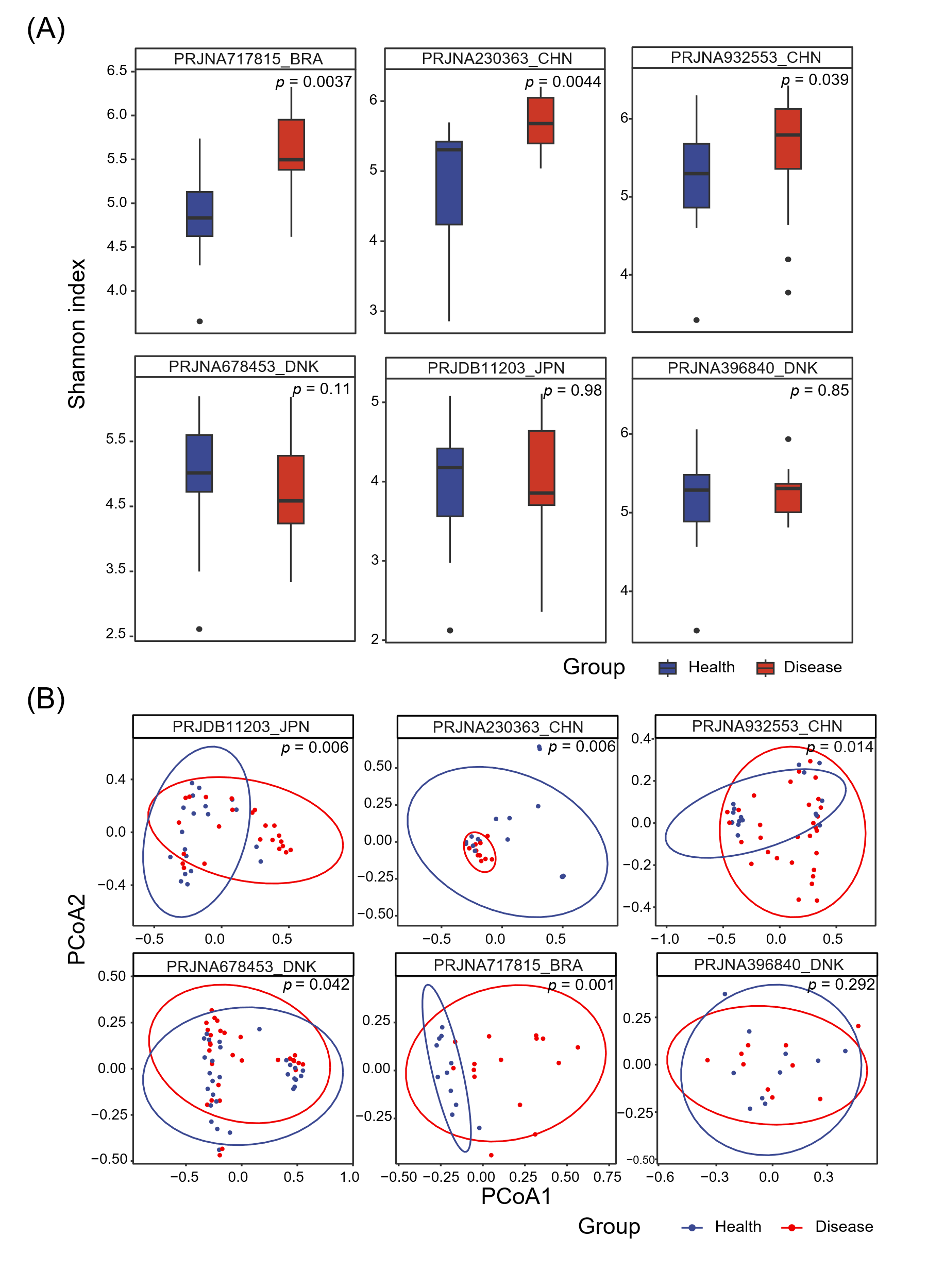
**

**Figure S2 Species diversity was analyzed based on taxonomic relative abundance data. (A)** Within-cohort alpha-diversity (Shannon index) comparison between disease and healthy groups in the six case-control cohorts. A two-sided Wilcoxon rank-sum test was used for group-wise comparisons. **(B)** Within-cohort beta-diversity analysis between disease and healthy groups using principal coordinates analysis (PCoA) in the six cohorts. *P* value was calculated with permutational multivariate analysis of variance (PERMANOVA) by 999 permutations (two-sided test). Each point is a sample.


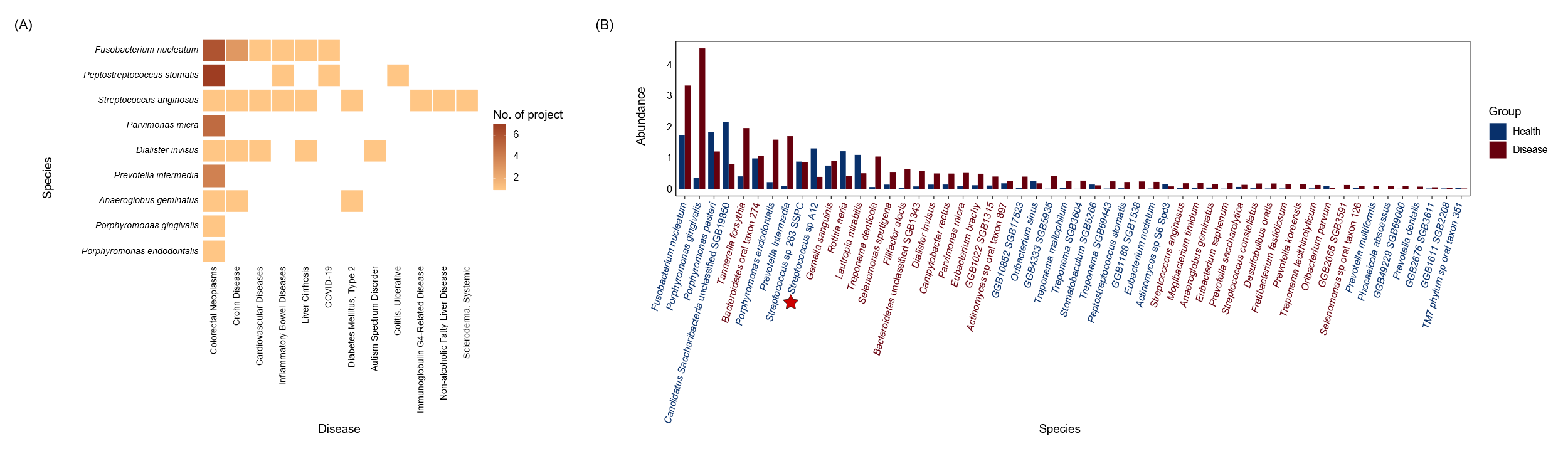


**Figure S3** **Disease type with biomarkers enriched as well as the abundance.** **(A)** The heatmap plot shows that the periodontitis oral biomarkers are also enriched in the gut microbiome of many diseases according to the GMrepo database. The color gradients indicate the number of projects of a particular disease in which the biomarkers are disease-enriched. **(B)** The bar graph compares the abundance of marker species in healthy and diseased samples, ranked from highest to lowest total abundance. The red bars represent diseased samples, and the blue bars represent healthy samples. The red font indicates disease-enriched marker species, and the blue font indicates health-enriched species.


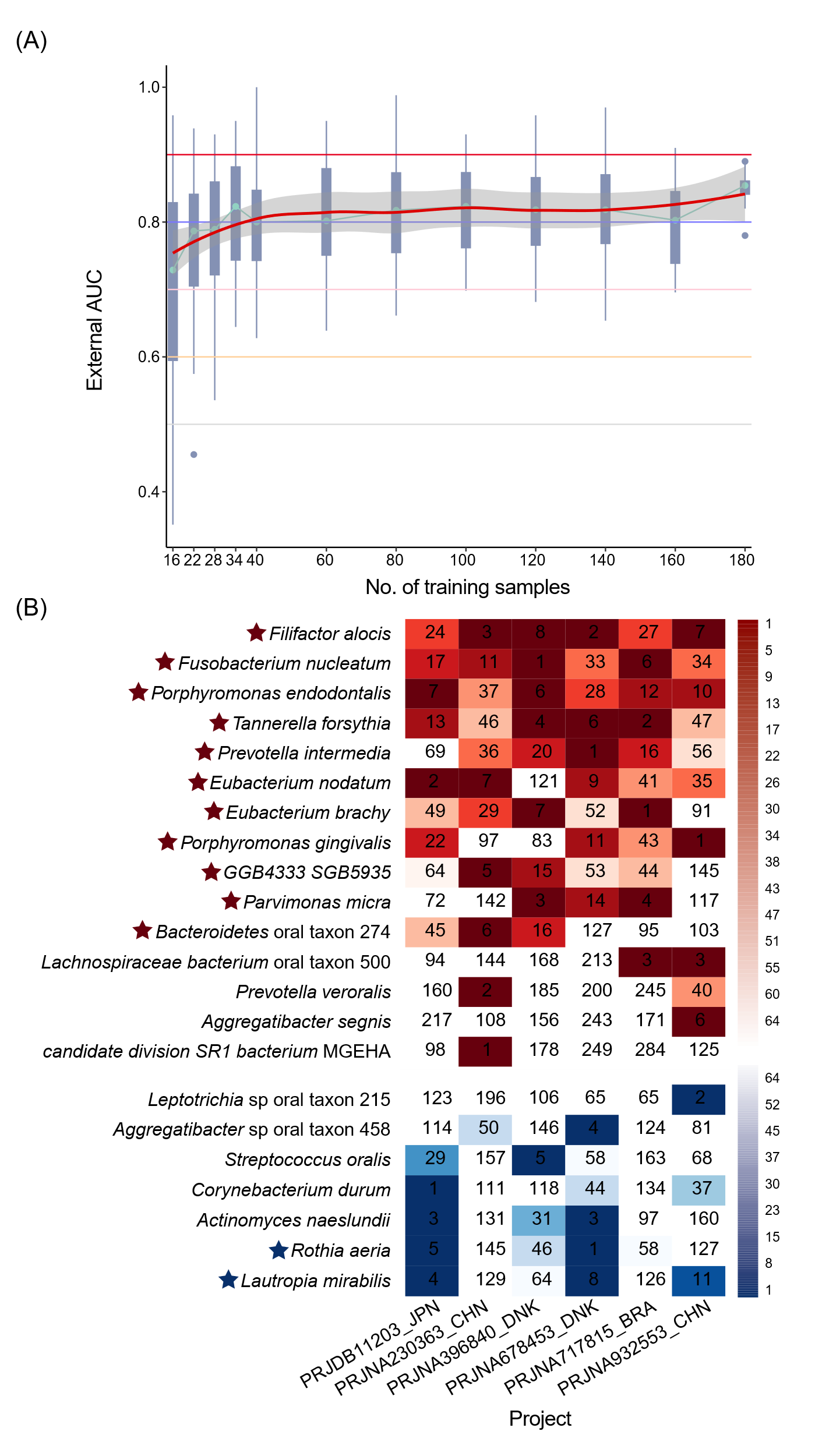


**Figure S4 Trends in external AUC with the number of training samples and heatmap of species ranked by RF classifiers. (A)** The box plot shows the trend of external AUCs as a function of the number of training samples, with the middle line representing the connection of the medians. **(B)** Heatmap showing species ranked by the RF classifiers. The top five features ranked by each of the six intra-cohort classifiers were selected. Case- and control-enriched features were ranked independently. The color gradient represents the mean rank of each feature. Red and blue stars represent marker species that are enriched in case and control groups according to the LEfSe analysis, respectively.


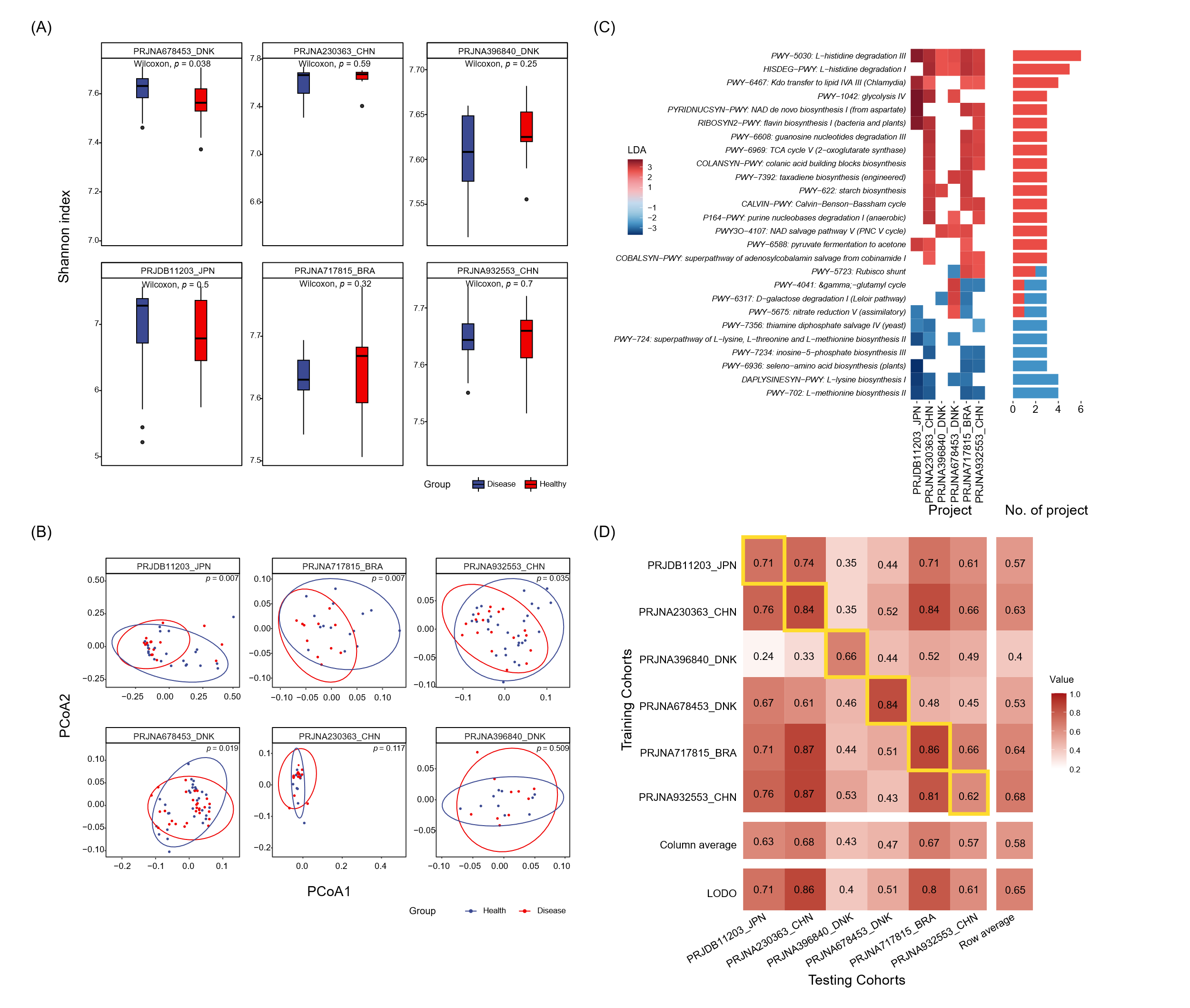


**Figure S5 Function-based diversity analysis, identification of cross-cohort pathway biomarkers, and modeling of machine learning classifiers for periodontitis. (A)** Boxplots show the alpha-diversity (Shannon index) of the six case-control cohorts using the functional profile. Significance was determined using the Wilcoxon rank-sum test (two-sided). **(B)** Within-cohort principal-component analysis (PCA) between the case and control groups in the six cohorts. P value was calculated with PERMANOVA by 999 permutations (two-sided test). **(C)** Heatmap-bar plot showing the metabolic pathway biomarkers that showed significantly differential abundances between case and control groups in more than three cohorts. The functional biomarkers were identified using LEfSE on HUMAnN3 pathway profiles; a linear discriminant analysis (LDA) score cutoff of 2 was used to select the biomarkers. The red and blue blocks indicate case- and control-enriched biomarkers, respectively. **(D)** Cross-prediction matrix reporting prediction performances as AUC values obtained using Random Forest (RF) classifiers on pathways-level relative abundances. The values boxed in yellow squares on the diagonal are the AUC values obtained by training and validating within individual cohorts. The non-diagonal values refer to training a classifier on the dataset corresponding to the row and applying it to the dataset corresponding to the column to obtain the AUC value. The "Row average" and "Column average" are the average AUCs of the corresponding rows and columns, excluding the diagonal values. The Leave-One-Dataset-Out (LODO) row refers to the performances obtained by training the model on the pathways-level abundances using all but the dataset of the corresponding column and applying it to the dataset of the corresponding column. Color gradient represents the value of AUC.

**
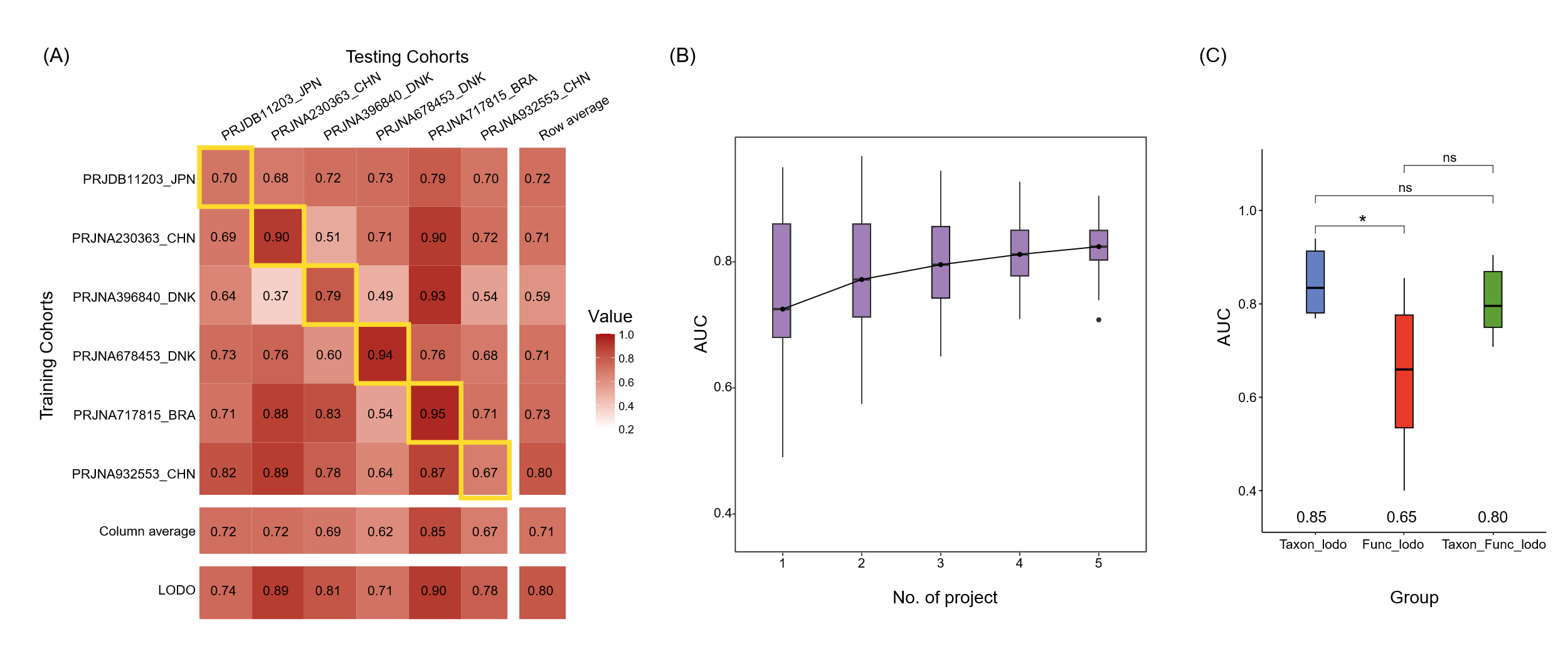
**

**Figure S6. Prediction performances of the models based on the combined taxonomic-functional profiles in within-cohort validation and cross-cohort testing. (A)** Cross-prediction matrix reporting prediction performances as AUC values obtained using a Random Forest (RF) model on species-pathways combined relative abundances. The values boxed in yellow squares on the diagonal are the AUC values obtained by training and validating within the queue. The non-diagonal values refer to training a classifier on the dataset corresponding to the row and applying it to the dataset corresponding to the column to obtain the AUC value. The row average and column average are the average values excluding the diagonal values. The Leave-One-Dataset-Out (LODO) row refers to the performances obtained by training the model on the species-pathways combined abundances using all but the dataset of the corresponding column and applying it to the dataset of the corresponding column. Color gradient represents the value of AUC. **(B)** Box plot showing the external AUC as the function of an increasing number of cohorts combined for model training. **(C)** The box plot shows the comparison of AUC values obtained from LODO analysis of taxonomic profiles, functional profiles, and combined taxonomic-functional profiles. Two sides Wilcoxon rank sum test was used for comparisons. The numbers at the bottom indicate the average AUC values corresponding to each model. **p* < 0.05, ***p* < 0.01, ****p* < 0.001, *****p* < 0.0001.


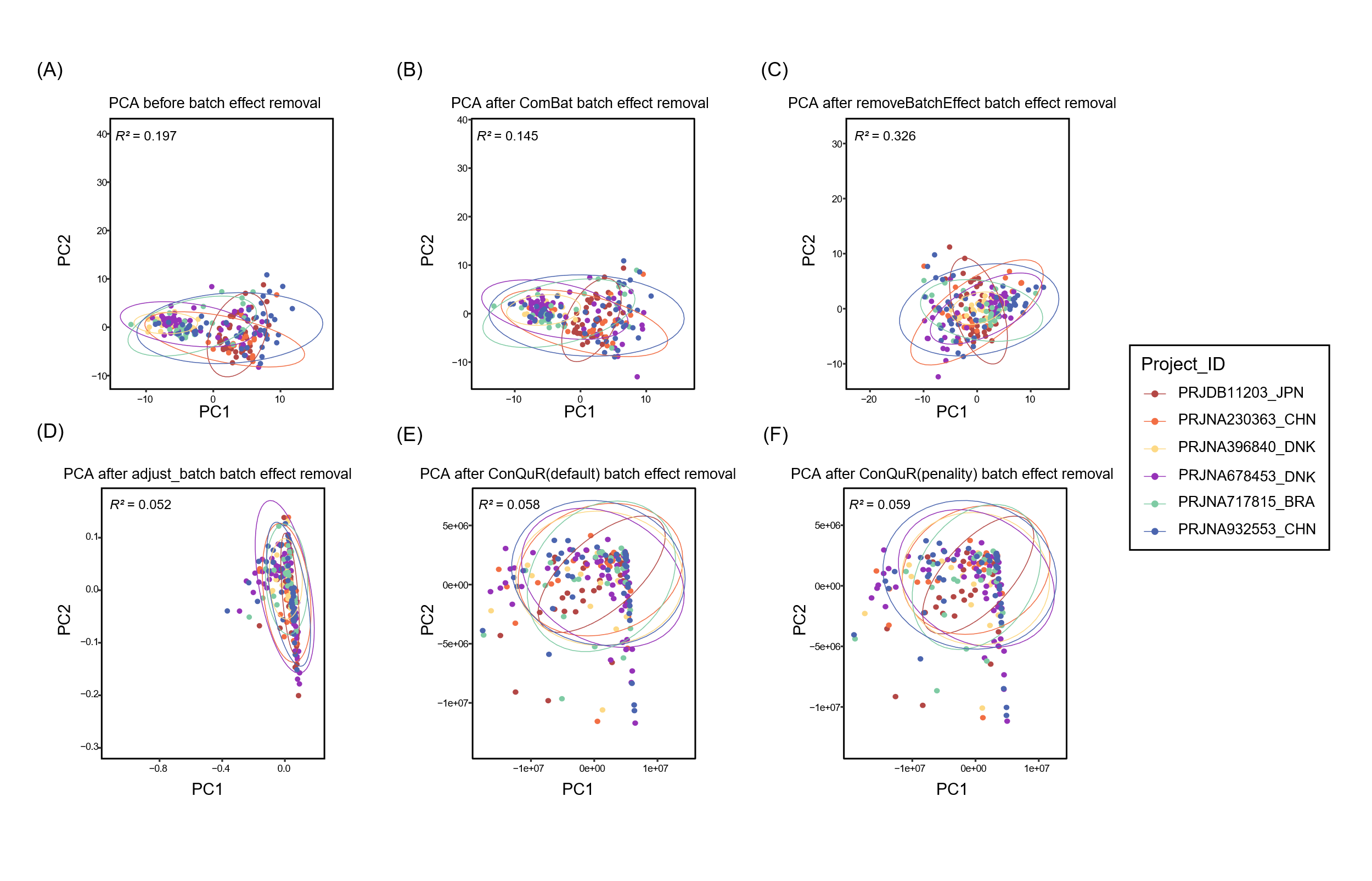


**Figure S7 Evaluation of batch-effect removal tools on the six case-control projects. Principal coordinates analysis (PCoA) plots showing the sample distribution before and after batch-effect removal using state-of-art tools, with the tool names indicated in the panel titles. (A)** sample distribution before any batch-effect removal tools were applied. **(B-F)** sample distributions after batch-effect removal tools were applied, including (B) “ComBat” function in the “sva” package, (C) “removeBatchEffect” in the “limma” package, (D) “adjust_batch” in the “MMUPHin” package, and I “ConQuR” in the “ConQuR” package with “default” parameter, and (F) “ConQuR” in the “ConQuR” package with “penality” parameter.


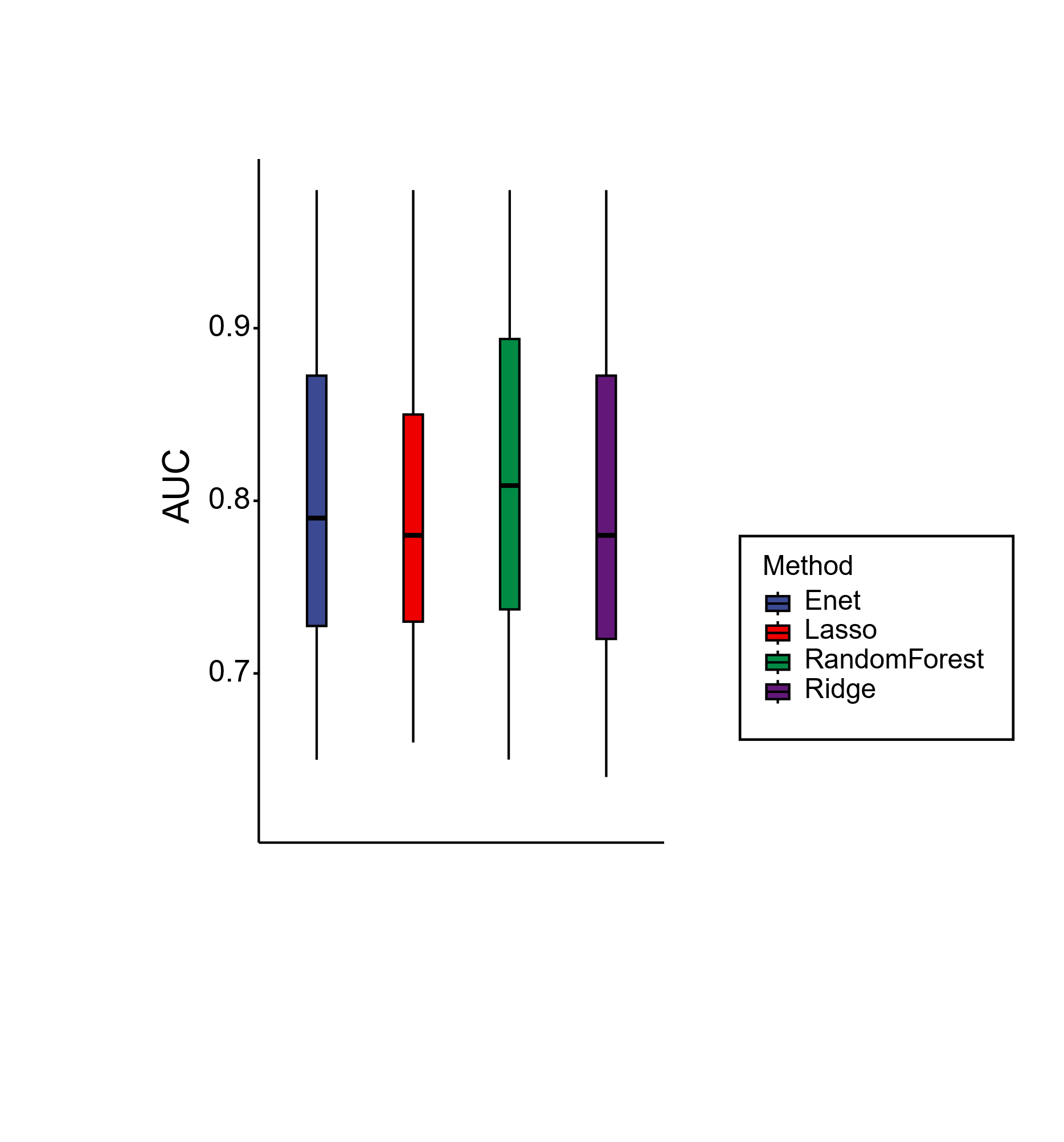


**Figure S8 Boxplot showing the performance of four machine learning algorithms in cross-validation.** The results of the random forest modeling were the best. We finally chose random forest as our machine learning algorithm.

# References

1. Katz, Kenneth, Oleg Shutov, Richard Lapoint, Michael Kimelman, J Rodney Brister, Christopher O'Sullivan. 2022. “The sequence read archive: a decade more of explosive growth.” *Nucleic Acids Research* 50: D387–D390. <https://doi.org/10.1093/nar/gkab1053>

2. Bolger, Anthony M, Marc Lohse, Bjoern Usadel. 2014. “Trimmomatic: a flexible trimmer for illumina sequence data.” *Bioinformatics* 30: 2114–2120. <https://doi.org/10.1093/bioinformatics/btu170>

3. Langmead, Ben, Steven L Salzberg. 2012. “Fast gapped-read alignment with bowtie 2.” *Nature Methods* 9: 357–359. <https://doi.org/10.1038/nmeth.1923>

4. Blanco-Míguez, Aitor, Francesco Beghini, Fabio Cumbo, Lauren J McIver, Kelsey N Thompson, Moreno Zolfo, Paolo Manghi, *et al*. 2023. “Extending and improving metagenomic taxonomic profiling with uncharacterized species using metaphlan 4.” *Nature Biotechnology* 41: 1633–1644. <https://doi.org/10.1038/s41587-023-01688-w>

5. Beghini, Francesco, Lauren J McIver, Aitor Blanco-Míguez, Leonard Dubois, Francesco Asnicar, Sagun Maharjan, Ana Mailyan, *et al*. 2021. “Integrating taxonomic, functional, and strain-level profiling of diverse microbial communities with biobakery 3.” *Elife* 10: <https://doi.org/10.7554/eLife.65088>

6. Li, Min, Jinxin Liu, Jiaying Zhu, Huarui Wang, Chuqing Sun, Na L. Gao, XingMing Zhao, Wei-Hua Chen. 2023. “Performance of gut microbiome as an independent diagnostic tool for 20 diseases: cross-cohort validation of machine-learning classifiers.” *Gut Microbes* 15: 2205386. <https://doi.org/10.1080/19490976.2023.2205386>

7. Nagata, Naoyoshi, Suguru Nishijima, Yasushi Kojima, Yuya Hisada, Koh Imbe, Tohru Miyoshi-Akiyama, Wataru Suda, *et al*. “Microbiome meta-analysis and cross-disease comparison enabled by the siamcat machine learning toolbox.”

8. Ritchie, Matthew E, Belinda Phipson, Di Wu, Yifang Hu, Charity W Law, Wei Shi, Gordon K Smyth. 2015. “Limma powers differential expression analyses for RNA-sequencing and microarray studies.” *Nucleic Acids Research* 43: e47. <https://doi.org/10.1093/nar/gkv007>

9. Leek, Jeffrey T, W Evan Johnson, Hilary S Parker, Andrew E Jaffe, John D Storey. 2012. “The sva package for removing batch effects and other unwanted variation in high-throughput experiments.” *Bioinformatics* 28: 882–883. <https://doi.org/10.1093/bioinformatics/bts034>

10. Ma, Siyuan, Dmitry Shungin, Himel Mallick, Melanie Schirmer, Long H Nguyen, Raivo Kolde, Eric Franzosa, *et al*. 2022. “Population structure discovery in meta-analyzed microbial communities and inflammatory bowel disease using mmuphin.” *Genome Biology* 23: 208. <https://doi.org/10.1186/s13059-022-02753-4>

11. Ling, Wodan, JiuyaoLu, Ni Zhao, AnjuLulla, AnnaM.Plantinga, Weijia Fu, Angela Zhang, *et al*. 2022. “Batch effects removal for microbiome data via conditional quantile regression.” *Nature Communications* 13: 5418. <https://doi.org/10.1038/s41467-022-33071-9>

12. Cao, Yang, Qingyang Dong, Dan Wang, Pengcheng Zhang, Ying Liu, Chao Niu. 2022. “Microbiomemarker: an r/bioconductor package for microbiome marker identification and visualization.” *Bioinformatics* 38: 4027–4029. <https://doi.org/10.1093/bioinformatics/btac438>

13. Zou, Hui, Trevor Hastie. 2005. “Regularization and variable selection via the elastic net.” *Journal of the Royal Statistical Society Series B: Statistical Methodology* 67: 301–320. <https://doi.org/10.1111/j.1467-9868.2005.00503.x>

14. Tibshirani, Robert. 2018. “Regression shrinkage and selection via the lasso.” *Journal of the Royal Statistical Society: Series B (Methodological)* 58: 267–288. <https://doi.org/10.1111/j.2517-6161.1996.tb02080.x>

15. Chen, Han, Nana Tang, Qiang Ye, Xin Yu, Ruoyun Yang, Hong Cheng, Guoxin Zhang, Xiaoying Zhou. 2022. “Alternation of the gut microbiota in metabolically healthy obesity: an integrated multiomics analysis.” *Front Cell Infect Microbiol* 12: 1012028. <https://doi.org/10.3389/fcimb.2022.1012028>

16. Goldstein, M., A. F. M. Smith. 2018. “Ridge‐type estimators for regression analysis.” *Journal of the Royal Statistical Society: Series B (Methodological)* 36: 284–291. <https://doi.org/10.1111/j.2517-6161.1974.tb01006.x>

17. Robin, Xavier, Natacha Turck, Alexandre Hainard, Natalia Tiberti, Frédérique Lisacek, Jean-Charles Sanchez, Markus Müller. 2011. “Proc: an open-source package for r and s+ to analyze and compare roc curves.” *BMC Bioinformatics* 12: <https://doi.org/10.1186/1471-2105-12-77>

18. Zhu, Jiaying, Chuqing Sun, Min Li, Guoru Hu, Xing-Ming Zhao, Wei-Hua Chen. 2023. “Compared to histamine-2 receptor antagonist, proton pump inhibitor induces stronger oral-to-gut microbial transmission and gut microbiome alterations: a randomised controlled trial.” *Gut* <https://doi.org/10.1136/gutjnl-2023-330168>

19. Wirbel, Jakob, Paul Theodor Py, Ece Kartal, Konrad Zych, Alireza Kashani, Alessio Milanese, Jonas S Fleck, *et al*. 2019. “Meta-analysis of fecal metagenomes reveals global microbial signatures that are specific for colorectal cancer.” *Nature Medicine* 25: 679–689. <https://doi.org/10.1038/s41591-019-0406-6>

20. Markus Riester, Wei Wei, Levi Waldron, Aedin C. Culhane, Lorenzo Trippa, Esther Oliva, Sung-hoon Kim, Franziska Michor, Curtis Huttenhower, Giovanni Parmigiani, Michael J. Birrer. Risk prediction for late-stage ovarian cancer by meta-analysis of 1525 patient samples. <https://doi.org/10.1093/jnci/dju048>

21. Oksanen, Jari, F. Guillaume Blanchet, Roeland Kindt, P. Legendre, R. G. O’Hara, Gavin Simpson, Peter Solymos, Hank Stevens, Helene Wagner. 2013. “Multivariate analysis of ecological communities in r: Vegan tutorial. R package version 1.7.”

22. Paradis, Emmanuel, Klaus Schliep. 2019. “Ape 5.0: an environment for modern phylogenetics and evolutionary analyses in r.” *Bioinformatics* 35: 526–528. <https://doi.org/10.1093/bioinformatics/bty633>
